# Tracing the evolution of chiropractic students’ confidence in clinical and patient communication skills during a clinical internship: a multi-methods study

**DOI:** 10.1186/1472-6920-12-42

**Published:** 2012-06-19

**Authors:** Mark Hecimovich, Simone Volet

**Affiliations:** 1 School of Chiropractic and Sports Science, Murdoch University, Murdoch, Western Australia; 2 School of Education, Murdoch University, Murdoch, Western Australia

## Abstract

**Background:**

Anecdotal evidence points to variations in individual students’ evolving confidence in clinical and patient communication skills during a clinical internship. A better understanding of the specific aspects of internships that contribute to increasing or decreasing confidence is needed to best support students during the clinical component of their study.

**Methods:**

A multi-method approach, combining two large-scale surveys with 269 students and three in-depth individual interviews with a sub-sample of 29 students, was used to investigate the evolution of change in student confidence during a 10-month long internship. Change in levels of confidence in patient communication and clinical skills was measured and relationship to demographic factors were explored. The interviews elicited students’ accounts and reflections on what affected the evolution of their confidence during the internship.

**Results:**

At the start of their internship, students were more confident in their patient communication skills than their clinical skills but prior experience was significantly related to confidence in both. Initial confidence in patient communication skills was also related to age and prior qualification but not gender whilst confidence in clinical skills was related to gender but not age or prior qualification. These influences were maintained over time. Overall, students’ levels of confidence in patient communication and clinical skills confidence increased significantly over the duration of the internship with evidence that change over time in these two aspects were inter-related. To explore how specific aspects of the internship contributed to changing levels of confidence, two extreme sub-groups of interviewees were identified, those with the least increase and those with the highest increase in professional confidence over time. A number of key factors affecting the development of confidence were identified, including among others, interactions with clinicians and patients, personal agency and maturing as a student clinician.

**Conclusion:**

This study provides insight into the factors perceived by students as affecting the development of professional confidence during internships. One particularly promising area for educational intervention may be the promotion of a pro-active approach to professional learning.

## Background

The internship is integral to most manual therapy programs, for example physical therapy, chiropractic, athletic training and osteopathy, and is often the first time students are exposed to real patients in a supervised, clinical situation. One of the main aims of the internship is to provide students with multiple opportunities to integrate theoretical knowledge with practical skills [1], while receiving ongoing guidance and feedback. Students are expected to record patient history, assess and treat patients, and provide counseling on a myriad of issues such as correct lifting and bending techniques, and exercise. Research shows that internships increase clinical competence, promote socialization within the profession, foster the development of a positive self-image, and promote professional confidence [2]. Furthermore, internships enable the collaboration of educators and mentors in facilitating the transition of the student to professional practitioner [3].

There is evidence to suggest that the internship is more significant than other learning opportunities, such as lectures and labs, in the building of confidence during a student’s tertiary experience, on the grounds that confidence increases with the experience gained through clinical exposure. Although this is well supported by the literature [4-7], there is evidence this may not always be the case. During the internship component students come face to face with inherent complexities of clinical practice, and the way in which these are navigated can vary significantly among students. For example, some students may possess high levels of perceived confidence prior to commencing their clinical rotation, only to find the reality of practice confronting and therefore experience a sudden reduction in confidence [8]. Alternatively, there may be peaks and troughs during internship, based upon continuous challenges that are sometimes successfully met but at other times lead to frustration [9]. Of particular concern for educators are the students who express confidence in procedures that they have had little experience in clinical settings [10]. Research on this phenomenon has highlighted the way in which students who are over-confident about their abilities can be a potential danger to patients [11].

To date, there is a paucity of empirical research in the development of confidence in clinical skills and patient communication in the field of manual and manipulative therapy. Research into the evolution and conditions of change in students’ professional confidence in manual medicine programs requires not only psychometrically reliable measures, but also measures with high levels of validity in a supervised clinical setting. Furthermore, in order to measure change in levels of confidence over a period of time, the instruments must be sensitive enough to capture the levels of change in confidence that can reasonably be expected during an internship. If one of the goals of the internship is to provide an environment that increases confidence alongside competence in clinical skills and patient communication, then developing a better understanding of how confidence evolves during the internship is imperative. Moreover, the factors that lead to either increased or decreased confidence need to be better understood.

This issue has been addressed by the study reported in this paper. The aims of the research were to: 1) measure initial and changing levels of confidence in students’ clinical and patient communication skills over the duration of an internship, with the consideration of the potential influence of gender, age, prior experience within the profession, and qualifications upon entry into the program; and 2) determine which specific aspects of the internship contributed to increasing or decreasing confidence in clinical skills and patient communication, from students’ own perspective.

## Methods

### Programs and participants

Participants (n = 269) were from seven different chiropractic programs, five located in the United States and two in Australia, enrolled in their clinical internship in 2006 and 2007. All programs had been selected due to similar curricula and clinical experience, which provided similar professional experiences for students. For example, the first two years consists of basic science units such as anatomy, biology, chemistry, physiology, biochemistry, immunology, genetics and microbiology. This followed by two to three years of clinical units such as biomechanics, nutrition, pharmacology, orthopedics, neurology, manipulative techniques, physical examination procedures, radiology and clinical internship. Human ethics approval and student consent were obtained from all cohorts before the study began.

### Procedures and instruments

A multi-method approach, combining two large-scale surveys (beginning and end of internship) and three in-depth individual interviews with a sub-sample of 29 students from a single cohort were used to investigate the evolution of change in student confidence in clinical skills and patient communication over the duration of the internship, and the factors that may influence the evolution of confidence during the internship, from students’ experiential perspective.

#### Instrument to measure confidence in clinical and patient communication skills

The instrument, developed by the authors, used [unpublished data under review, Hecimovich, Styles, Volet] for measuring self-assessed confidence in these two aspects of professional confidence consists of two scales, each capturing the multi-faceted nature of the target construct:

1. Patient Communication Confidence Scale (PCCS): 28 items

2. Clinical Skills Confidence Scale (CSCS): 27 items

Demographic information regarding age, gender, prior relevant experience, and level of qualification upon entry into the program was also collected, to investigate predictors in initial levels of confidence, and possible impact on further development of confidence.

The PCCS and CSCS are customized to the supervised clinical setting, and therefore measure students’ self-assessed levels of confidence in patient communication and clinical skills in relation to interactions with real patients during the clinical internship. The items presented as questions, are formulated to capture real-life experiences students are most likely to encounter with patients during their internship. Issues range from general to specific, for example, from how confident students felt in discussing general health issues with patients to performing basic and very specific physical examination procedures. Sample items include, “How confident are you in your ability at discussing personal and/or sensitive issues with new patients?” and, “How confident are you in your ability to perform basic physical examination procedures such as blood pressure, pulse and respiration rates on a patient?” Response categories were presented in a six-point Likert-style response format from 1 to 6, from “not confident at all” to “very confident”. There are no reverse items.

The psychometric properties of the two scales PCCS and CSCS were established through comprehensive psychometric analysis based on the Rasch measurement model [12,13], using the software program, RUMM2020 developed by Andrich et al [14]. The two scales were found to be valid and reliable measures of confidence with high Person Separation Indices (0.96 for PCCS; 0.93 for SCSC), which are equivalent of Cronbach’s alpha statistics. Through the Rasch analyses, each scale is standardized with a mean of zero, thus the generation of positive and negative scores for individuals. Data from beginning and end surveys were analysed together to obtain beginning and end scores based on the same hierarchical response scale.

To address validity, two existing valid and reliable scales - the General Self-Efficacy scale (GSE) and the Personal Report of Communication Apprehension scale (PRCA-24) - were included in the battery of tests. The GSE scale measures feelings of mastery in various situations, providing evidence of generalised self-efficacy among students. Previous research shows the GSE is a reliable scale with convergent and discriminant validity, with alpha reliability coefficients ranging from .75 to .90 [15,16]. The PRCA-24 scale measures feelings about communicating with others. However, only one sub-category (interpersonal communication) was used in this study, as the other sub-categories are not typically encountered in clinical contexts. Prior research has demonstrated content, criterion and construct validity of the PRCA-24 [17]. Results showed a positive correlation between confidence scores for clinical and patient communication skills and general self-efficacy, and a negative correlation between confidence scores for patient communication and interpersonal communication, indicating significant associations between the new and existing scales and add to the evidence of the validity of the new scales.

Analyses of variance (ANOVAs) were used to examine the extent to which students’ initial levels of confidence in clinical and patient communication skills were related to age, gender, prior experience or possession of a first degree, with possible interaction between factors. Change in levels of confidence in clinical and communication skills was investigated using multiple analyses of variance (MANOVA) with repeated measures for time.

#### Interviews

In addition to completing the beginning and end of internship surveys, 29 students from the same program gave their consent to participate in three semi-structured individual interviews, conducted at regular intervals over the 10-month duration of their internship: stage 1 at months one or two; stage 2 at months five or six; and stage 3 at months nine or ten. These interviews were conducted in an informal, conversational style. The main purpose was to elicit students’ reflections on what may have contributed to increasing or decreasing their confidence during their internship. An example of question to prompt reflection was, “Has there been anything recently which has helped or hindered your confidence in being able to communicate with a patient?”, which was followed up by probes to obtain more in depth responses. All interviews were transcribed.

A thematic analysis of the interview data was undertaken to identify key factors that impact self-confidence in patient communication and clinical skills. Thematic analysis is delineated as a qualitative analytic method that is utilized for identifying, analyzing and reporting themes with data [18]. It comprises several phases. The first phase consisted of reviewing the sets of transcripts after the interviews several times and observing for meanings and emerging patterns. The second phase involved coding and the production of initial (open) codes. The third phase involved the process of axial coding, whereby all data was initially coded, a list of different codes was developed, these different codes were sorted into potential themes, and relevant extracts of coded data were then collated under the identified themes. The fourth and fifth phase involved reviewing, refining and naming the different themes that were identified during initial (open) and axial coding, and then developing a ‘thematic map’ and assessing whether it reflected the meanings evident in the data set as a whole. In this research, the ‘thematic map’ contained the various themes that affected student professional self-confidence and emphasized aspects of these themes that were helpful or a hindrance.

## Results

### Confidence in clinical and patient communication skills prior to internship, overall and in relation to demographic factors

Overall students appeared more confident in their patient communication skills (PC, M = 1.31, SD = 1.87) than in their clinical skills (CS, M = 0.55, SD = 1.08) before starting the internship. The correlation coefficient between the two scales was r = .68 (p < .001), showing that confidence in patient communication and confidence in clinical skills were inter-related, and may form an overarching construct of professional confidence.

The extent to which the demographic factors were related to initial levels of confidence was examined separately for patient communication and clinical skills. Table 1 displays the breakdown in levels of confidence by gender, age, prior experience and prior qualification for patient communication (PC). As can be seen, confidence in patient communication was significantly related to age, prior experience and prior qualification but not to gender. While the relationship with prior experience and prior qualification would be expected in the area of patient communication, the relationship with age was not expected. Comparing the three age groups shows that confidence increased as age increased. All tests statistics are displayed on the right hand side of the table and the results of post-hoc comparisons using Bonferroni are at the bottom.

**Table 1:** Initial confidence in patient communication skills broken down by demographic factors

**Factor**	**Group**	**N**	**Mean(std dev)**	***F*statistic**	***p*value**
Gender	Male	153	1.38 (1.88)	.598	p = .44
	Female	116	1.21 (1.75)		
Age	20-25	136	0.97 (1.50)	4.934	p = .008
	26-35	106	1.63 (1.95)		
	36+	27	1.78 (2.43)		
Experience	None at all	48	1.19 (2.00)	3.626	p = .003
		63	0.71 (1.19)		
		56	1.11 (1.83)		
		54	1.71 (1.90)		
		25	2.10 (2.14)		
	Extensive	23	1.90 (1.77)		
Qualification	Possess degree upon entry	153	1.53 (1.93)	5.16	p = .024
	No degree upon entry	116	1.02 (1.64)		

Post-hoc comparisons by Age using Bonferroni revealed a significant difference (*p =* .017) between the 20–25 and 26–35 age groups. Post-hoc comparison by Experience using Bonferroni revealed a significant difference between level 2 and level 4 (*p* = .044) and level 5 (*p* = .016).

Table 2 displays the breakdown in initial levels of confidence for the same demographic factors for clinical skills (CS). As expected and like for patient communication, prior experience was significantly related to levels of confidence in clinical skills. However, and in contrast to patient communication, there was no relationship with age or prior qualification, suggesting that communication skills may be transferable across situations whereas clinical skills are not. Results of the statistical analyses and post-hoc comparisons using Bonferroni are showed respectively on the right hand side and at the bottom of the table.

**Table 2:** Initial confidence clinical skills broken down by demographic factors

**Factor**	**Group**	**N**	**Mean(std dev)**	***F*statistic**	***p*value**
Gender	Male	153	0.31 (1.04)	4.839	p = .029
	Female	116	0.03 (1.05)		
Age	20-25	136	0.11 (0.95)	.861	p = .424
	26-36	106	0.29 (1.19)		
	36+	27	0.18 (0.99)		
Experience	None at all	48	0.12 (1.08)	2.639	p = .024
		63	−0.06 (0.89)		
		56	0.02 (0.96)		
		54	0.44 (1.15)		
		25	0.45 (1.28)		
	Extensive	23	0.58 (0.92)		
Qualification	Possess degree upon entry	153	0.27 (1.12)	1.910	p = .168
	No degree upon entry	116	0.09 (0.89)		

Post-hoc comparison by Experience using Bonferroni revealed none had significant difference.

### Change in confidence in patient communication and clinical skills over the duration of the internship

Students’ levels of confidence in patient communication (PC) skills and clinical skills (CS) increased significantly over the duration of the internship. The means, standard deviation, and results of paired t-tests for both patient communication (PC) and clinical skills (CS) are displayed in Table 3.

**Table 3:** Means, standard deviation and results of paired t-tests for both patient communication (PC) and clinical skills (CS) at the beginning and end of the clinical internship

	**Beginning of clinical internship**	**End of clinical internship**	**Paired t-tests**
	**Mean (std dev)**	**Mean (std dev)**	***p*value**
Patient communication skills	1.21 (1.77)	2.72 (1.92)	t(207) 13.86, p < .001
Clinical skills	0.06 (0.99)	1.12 (1.18)	t(207) 15.07, p < .001

To determine if change in levels of confidence in patient communication and clinical skills were related to each other, ‘difference’ scores were computed, for both measures and correlated. The high correlation coefficient between the two change measures, r = 0.72, p < .001 showed that change over time in these two aspects of professional confidence were inter-related.

The extent to which change over time in PC and CS was influenced by demographic factors was examined next. A series of multiple analyses of variance (MANOVA) with repeated measures for time were carried out, with in turn experience, age, gender and qualification as the independent variable. Table 4 displays the results of these analyses.

**Table 4:** MANOVA Main effects for time, age, gender and qualification

**MANOVA analysis**	**1 vs 3 (n = 208) 10 month internship**		
**Category**	**Interaction effect**	**Main for time as (repeated factor)**	**Main for experience, age, gender or qualification**
*Experience by time*			
PCCS	p = .329	p = .000	p = .000
CSCS	p = .580	p = .000	p = .008
*Age by time*			
PCCS	p = .331	p = .000	p = .003
CSCS	p = .264	p = .000	p = .219
*Gender by time*			
PCCS	p = .476	p = .000	p = .242
CSCS	p = .962	p = .000	p = .001
*Qualification by time*			
PCCS	p = .702	p = .000	p = .038
CSCS	p = .657	p = .000	p = .145

As can be seen in Table 4, no interaction effects were found between any of the independent variable and time as the repeated measure. A number of significant main effects were found for the four independent variables, all of them consistent with the analyses conducted at the start of the internship. In other words, all the demographic factors identified as influencing students’ levels of confidence in PC and CS at the beginning of the internship were found to be significant in students’ further development of professional confidence over time.

While the enduring influence of prior experience, and to some extent qualification and age, on confidence development in patient communication skills could be expected, the sustained gender differences on the development of confidence in clinical skills deserve some attention.

Although overall levels of confidence increased significantly over the duration of the internship for both patient communication and clinical skills, there were large individual differences in the degree of increase. For some students, only a limited increase was observed while others displayed a dramatic increase. Possible reasons for these differences were explored in the interviews.

### Interviews

The purpose of the interviews was to determine which specific aspects of the internship contributed to increasing or decreasing confidence in clinical skills and patient communication throughout the internship, from students’ own perspective. For a synopsis of the aspects, please consult Table 5. To explore this issue, two extreme sub-groups of students from the same instructional context (n = 106) were identified on a normative basis determined by the questionnaire data: a sub-group displaying the greatest increase (PC: 2.77 - 4.8; CS: 1.79 - 1.98) and a sub-group displaying the least increase (PC: -0.3 - 1.02; CS: .10 - 1.18) in confidence. Using this normative approach, students were categorized in the high increase category if their score on PC or CS increased from being located in the lower to mid 33 % at the beginning of the internship to the upper 33 % at the end of the internship, and reciprocally, in the low increase category if their score on PC or CS went from the upper to the middle 33 % at the beginning of the internship to the lower 33 % at the end.

**Table 5:** Synopsis of factors influencing the evolution of confidence in clinical skills and patient communication

**Factors and stages in which they appeared**	
**Clinical skills**	**Patient communication**
*Interaction with clinicians* (stages 1, 2, 3)	*Meet and greet the patient (stage 1)*
A help and hindrance with verbal (positive and negative comments) and non-verbal (demonstrating skill) feedback being key elements	Allowing students to initiate meeting and greeting the patient provides them to make a quick and helpful initial assessment
*Audible noise* (stage 1, 2, 3)	*Interaction with clinicians* (stage 1, 2, 3)
Students relying on this as an indicator of success; forgoing other indicators such as pain, range of motion, activities of daily living	*Non-challenging conditions* (stage 1, 2, 3)
*Perceived limited skills* (stage 2, 3)	Patient conditions which do not challenge the student limits their ability to mature
Linked with lack of audible obtained with manipulation	*Personal agency* (stage 2, 3)
*Personal agency* (stage 3)	*Maturing as a student clinician* (stage 2, 3)
Students seeking way to improve is an indicator of maturity	Building rapport with patients and recognizing why they seek care
	*Patient conflicts* (stage 3)
	Lack of maturity and personal agency result in poor patient encounters
	*Perceptions of the profession* (stage 3)
	Questioning the profession

Of the 106 students, 24 displayed a high or a low increase in confidence in either PC or SC or both. Of these 24 students, 12 had been interviewed. By chance six students fell into the high increase category, PC (n = 3) or CS (n = 2) or both (n = 1), and six students into the low increase category, PC (n = 3) or CS (n = 0) or both (n = 3). Hereafter, students are identified as Low Incr PC, Low Incr CS, High Incr PC, High Incr CS, preceded by their research id, and followed by the stage of their internship and page number of the interview transcript.

#### Key factors influencing the development of confidence in clinical skills

Although the researchers’ intent was to gain a better understanding of the factors, influencing confidence in a broad range of clinical skills, including physical examination and manipulative procedures, it turned out to be the manipulative procedures, or adjustments, which students mainly focused upon. Four key factors were identified: Encounters with clinicians; audible noise; perceived poor skills, and; personal agency.

1. Interaction with cliniciansEncounters and the interaction with clinicians, a significant factor for all students in both groups was found to be a central issue for students during stage 1 interviews. Clinician feedback was reported as a key element in their encounters with clinicians.Their influence continued to be a factor as the students progressed through the internship, with feedback still being a key element, *What I have found really helpful is the clinician really saying, “OK. You need to do this.” “You need to take a more lateral flexion.” Or see me setting up on them (but) it just hasn’t translated into my confidence levels.* (8, High Incr CS, stage 2, p5). *It would be feedback from clinicians, because it seems as they’re the one that’s sort of assessing or marking or supervising us and whenever I get a good feedback from a clinician I would feel as though I’ve improved and that would increase my confidence.* (10, High Incr CS,, stage 3, p12). However, during the later stages personality issues were also being brought forth.

2. Audible noiseAnother influencing factor (positive or negative) identified in the first few months was the audible, popping or cracking, noise given off during a manipulative procedure, which students used as a way to measure success in the skill. Interestingly, only those whose confidence rose significantly identified this during the first interview stage.This factor became more central for both groups at the time of the stage 2 interviews, *a lack of experience of achieving a cavitation in that joint. Lack of success* (8, High Incr CS, stage 2, p4). One student specified that while other outcomes such as reduction of pain were important, the audible remained the key element of success, *I like the patient out of pain but I guess hearing the release* (audible) *is probably the most gratifying.* (11, High Incr CS, stage 2, p13). *… success rate…*(How do you measure success?) *If you get an audible.* (6, Low Incr CS, stage 2, p21).Towards the end of the internship, only students with limited increase in confidence identified the audible as a factor. The placebo effect of the audible was perceived as beneficial for them and the patient.

3. Perceived limited skillsAs students progressed through the internship, those with limited increase in confidence perceived their limited manipulation skills as a hindrance to confidence. This perception lead them to doubting themselves in the latter half of the internship.

4. Personal agencyIn contrast, the latter half of the internship saw those who had significant increase in confidence in manipulative skills become more proactive. They displayed personal agency by seeking ways to improve their skills and a mature approach to perceived failure. *I’ve been asking around for more people to correct me and going to the technique lab. This has helped* (10, High Incr CS, stage 3, p8).In contrast, those students with limited increase in confidence also reflected on the influence of the clinician but instead of seeking ways to improve, they displayed self doubt which inhibited taking action.

#### Patient communication

Various factors influenced student confidence in patient communication as they progressed though the internship. Similar to the reflections on clinical skills, the clinicians played a major role in their confidence. Those students whose confidence rose significantly became more proactive and matured as clinicians while the other group struggled with clinician and patient conflicts and with the profession’s identity. Seven factors were identified as influencing confidence in patient communication.

1. Meet and greet the patientMeeting and greeting the patient in the waiting room, as opposed to the clinician doing this, was identified as a factor in the early stages. For students, this helped them understand the patient’s condition and therefore boosted confidence. *Because you can see how they rise from a chair and you can kind of do a very quick initial assessment* (7, High Incr PC, stage 1, p9).

2. Interactions with the cliniciansThe nature of the interactions between student and clinician was brought forth as a significant factor in confidence for all students. These interactions contributed to boosting or hindering confidence in the early stages on the internship.As the internship progressed, those whose confidence rose significantly relied less on the clinicians, but the other group continued to reflect on how interaction with the clinicians affected their confidence in a positive way, *to hear that from a clinician made a difference…but coming from a clinician boosts your confidence more* (6, Low Incr PC, stage 2, p3-4), but also in a negative way.The significance of clinician interactions continued to be a factor within this group even into the latter stages of the internship.

3. Non-challenging patient conditionsStudents with limited increase in confidence identified non-challenging patient conditions as being a confidence boost in the early stages of the internship, *If it’s musculoskeletal I feel quite confident. But if it comes to something else, say stomach or thyroid, it drops* (2, Low Incr PC, stage 1, p3). … *it comes down to what the complaint is, if it’s easy to explain. If it’s hard to explain then you get more worried.***(**6, Low Incr PC, stage1, p20)As students progressed through the internship, difficult cases challenged their confidence in communicating with patients, *I haven’t had anything that has been too curly if you know what I mean…so that may well change when I get a patient come through that’s maybe got something a little more serious.*.*…if a patient came in and I found out the prognosis wasn’t so good, that may affect my communication right there and then…therefore affect my confidence as well* (5, Low Incr PC, stage 2, p2-3). By the latter stages this continued, *It is not like I wouldn’t be able to communicate but my confidence level would be a 6/6 of mechanical low back and maybe 3/6 for a strange arthridity. So there’s an influencing factor on confidence* (2, Low Incr PC, stage 3, p7)

4. Personal agencyApproximately five to six months into the internship students who had significant increases in patient communication confidence displayed proactive ways of improving their communication skills.

5. Maturing as a clinicianThose students whose confidence rose significantly and had developed personal agency also started to mature as clinicians. What helped boost their confidence was their ability to build rapport with their patients, *I think it (confidence) has gotten a little bit higher because I’ve built a good rapport with my patients, I tend to remember what they did, like what they were telling me that they were going to do on the weekend, so I will ask them what they did on the weekend when they came in.* (7, High Incr PC, stage 2, p13).These students also recognized patient action and interaction as a significant boost in their confidence to communicate.

6. Patient conflictsIn contrast, many students with limited increase in patient communication confidence expressed personality conflicts with patient during the latter stages of their internship, *(My confidence in patient communication is) Less with new patients because you don’t know what their personality is, some patients are quite standoffish* (3, Low Incr PC, stage 3, p2). Some projected blame onto the patient, *something has hindered (my confidence) but that’s based on a patient. I found it very difficult to control the history. I’d ask a question and I would be there for the next ten minutes listening and I’d try an jump in there and you know I’d try and take control and he just wouldn’t stop.* (6, Low Incr PC, stage 3, p16).

7. Perceptions of the professionSome aspects of the chiropractic profession were found difficult for students with a limited confidence increase. While this emerged only in the latter stage of the internship, it seemed to affect some students’ ability to confidently communicate with patients.

The factors identified by students as influencing the evolution of their confidence in patient communication and clinical skills are further discussed below, with some educational implications.

## Discussion

One of the aims of the study was to identify how levels of confidence in students’ clinical and patient communications skills evolved over the duration of an internship in relation to selected demographic factors. The survey data revealed that prior experience was significantly related to initial and evolving levels of confidence in both patient communication and clinical skills. In contrast, gender, age and prior qualification were related to the development of either clinical or patient communication skills.

The relationship between initial confidence and prior experience was expected, and is consistent with Schunk’s [19] claims that exposure to models can instill self-beliefs that influence individuals’ subsequent course of action. Yet the finding that the sub-group of students with no prior experience displayed higher levels of confidence than those with moderate experience may indicate overconfidence, a concerning phenomenon which is discussed extensively in the literature [20].

The finding that male students rated their confidence in clinical skills higher than female students is consistent with evidence of females tending to underestimate their abilities [21] while their male counterparts overestimate their abilities [22]. This is consistent with those found in other fields, such as confidence in physics and computing [23,24]. According to Beyer & Bowden [21], confidence may differ between genders due to the type of skill or task involved. They found that females consider themselves equally competent (high level confidence) when tasks are perceived as gender-neutral or (traditionally) ‘feminine-type’ skills (e.g. verbal or interpersonal) and underestimate their abilities in (traditionally) ‘masculine-type’ skills or occupations. This was demonstrated in studies with medical students [25-27], where female students underestimated their surgical skills, possibly due, according to the researchers, to the perceived notion of surgeon as a (traditionally) ‘masculine’ occupation. A similar perception may have contributed in explaining the findings in the present study. Some of the clinical skills utilized in manual medicine, such as joint manipulation, possibly being perceived as more physical and therefore ‘masculine’.

The significant relationship between age or a prior degree and initial levels of confidence in patient communication skills, may indicate that interpersonal communication is a generic skill acquired through life experience and transferable to professional situations. In contrast, the lack of relationship between these two factors and confidence in clinical skills is consistent with those skills being highly specific to the profession. Interestingly, the sub-group of students in the highest age category tended to display lower levels of confidence in clinical skills than those in the mid age range. This might be due the myriad of issues that mature-age students face in their lives, which can lead to erosion in their confidence [28,29]. For example, in the field of health education, Feil et al [30] reported older medical students’ sense of loss of previous personal and professional identity, and Donaldson and Graham [31] reported older students’ frequent admission that they possess ‘rusty study skills’, low self-confidence and apprehension upon re-entering college.

The present study also aimed to examine the factors perceived by students as contributing to increase or decrease their confidence over time. The interview data with sub-groups of students, who demonstrated either a limited or a substantial increase in confidence, provided insight into the evolution of their confidence during the internship.

Interaction with clinicians and clinician feedback emerged as a prominent factor in the evolution of clinical and patient communication skills. The criticality of constructive feedback is well documented in the mentoring literature [32,33]. In the study, negative feedback was primarily reported as being in the form of verbal comments in which the students perceived the clinicians as putting them down and not treating them as peers, or taking over for the student and treating the patient. In contrast, positive feedback was verbal and non-verbal with clinicians providing supporting comments but also demonstrating how to perform a skill. This is consistent with Pitney and Ehlers [34] who stressed how mentor accessibility, approachability, and student initiative need to be enforced as these create an environment encouraging student participation and assist in the building of confidence [35].

Over time, students with high increases in confidence relied less on the clinicians while the other group continued to have mixed relationships with them. Some admitted relying too much on them while others still perceived them as being harsh in their approach. Interestingly, students displaying higher increases in confidence appeared more proactive and mature as student-clinicians, resulting in them relying less on clinician support. In contrast, those with low increases had not yet matured as student-clinicians. They continuously relied on clinicians, struggled with challenging patient conditions and patient conflicts and perceived limited skills. This is consistent with Bandura’s [36] claim that self-efficacy, or confidence, is the foundation of human agency, since unless people believe they can produce desired effects by their actions, they have little incentive to act. This may explain why in this study, one sub-group had little incentive to seek ways to improve. More research is needed to fully understand this phenomenon.

A factor which was brought up by both sub-groups was the importance of the audible noise released from a manipulative procedure, as evidence of successful adjustment and with a direct effect on confidence, has received limited attention in the literature. Yet, according to Flynn et al [37]*,* and Cleland et al [38], a joint audible can have a powerful placebo effect on both the patient and practitioner. This was demonstrated in this study, with some students attributing even more importance to the audible noise than patient outcomes as a measure of success. However, even if students focused on patient outcomes such as pain, range of motion and daily living activities, and minimized their confidence in the audible, they would still need to contend with the effect it has on patients. When their confidence is limited, they may struggle to communicate to their patients that the audible is not a representation of success.

Finally, some students’ reflections on the chiropractic profession raises the broader issue of how students perceive their chosen profession and in this instance, chiropractic. However, the fact that only students with limited increases in confidence addressed this issue is noteworthy. A myriad of reasons may explain such perceptions but recent research involving non-practicing chiropractors [39] has pointed to chiropractic dogma and philosophy as reasons to abandon active practice.

There were a number of limitations that warrant attention. First, focusing on and measuring students in the chiropractic field may not be generalized to the full range of manual medicine programs, also including osteopathy, physiotherapy and athletic training fields. Second, the contextual scope of the qualitative (interview) data was limited to two separate student cohorts from a single university chiropractic program. These factors make it difficult to generalize the findings to other contexts and student experiences. Due to the small sample size, this study could not reliably relate interview findings and demographic factors, and therefore identify tendencies. Yet, such connections may exist and could be examined in future research. Also the research did not compare the impact of different clinical opportunities, such as the on-campus internship and the external placement.

This study revealed that such perceptions could evolve as early as during professional education.

## Conclusion

The rich data that emerged from this multi-method study may assist clinical educators in manual medicine programs develop means of measuring professional confidence and fostering its development. One particularly promising area for educational intervention may be the promotion of a pro-active approach to professional learning.

## Competing interests

The authors declare that they have no competing interests.

## Authors’ contributions

MH conducted the individual interviews. MH and SV performed the content analysis. All authors contributed substantially to the conception and design of the study, as well as to the critical revision of the paper. All authors approved the final manuscript.

## Funding

There was no funding for this project.

## Pre-publication history

The pre-publication history for this paper can be accessed here:

http://www.biomedcentral.com/1472-6920/12/42/prepub
